# DeepmRNALoc: A Novel Predictor of Eukaryotic mRNA Subcellular Localization Based on Deep Learning

**DOI:** 10.3390/molecules28052284

**Published:** 2023-03-01

**Authors:** Shihang Wang, Zhehan Shen, Taigang Liu, Wei Long, Linhua Jiang, Sihua Peng

**Affiliations:** 1 School of Information Engineering, Huzhou University, Huzhou 313000, China; 2 Shanghai Institute for Advanced Immunochemical Studies, School of Life Science and Technology, ShanghaiTech University, Shanghai 201210, China; 3 College of Fisheries and Life Science, Shanghai Ocean University, Shanghai 201306, China; 4 Department of Radiology, Ruijin Hospital, Shanghai Jiao Tong University School of Medicine, Shanghai 200025, China; 5 College of Information Technology, Shanghai Ocean University, Shanghai 201306, China; 6 European Institute of Innovation and Management, Universitat Politècnica de València, 46022 Valencia, Spain

**Keywords:** mRNA subcellular localization, artificial intelligence, chaos-game representation, deep learning

## Abstract

The subcellular localization of messenger RNA (mRNA) precisely controls where protein products are synthesized and where they function. However, obtaining an mRNA’s subcellular localization through wet-lab experiments is time-consuming and expensive, and many existing mRNA subcellular localization prediction algorithms need to be improved. In this study, a deep neural network-based eukaryotic mRNA subcellular location prediction method, DeepmRNALoc, was proposed, utilizing a two-stage feature extraction strategy that featured bimodal information splitting and fusing for the first stage and a VGGNet-like CNN module for the second stage. The five-fold cross-validation accuracies of DeepmRNALoc in the cytoplasm, endoplasmic reticulum, extracellular region, mitochondria, and nucleus were 0.895, 0.594, 0.308, 0.944, and 0.865, respectively, demonstrating that it outperforms existing models and techniques.

## 1. Introduction

Eukaryotic mRNAs can be transported to various locations within the cell. The distribution of mRNA follows the organism’s programmed mechanism, and mRNAs are not evenly distributed in various subcellular locations [1,2,3,4]. The mRNA subcellular localization reveals the temporal and spatial characteristics of gene expression [5]. Incorrect mRNA localization can cause a variety of disorders, such as spinal muscular atrophy, Alzheimer’s disease, and various cancers [6]. Elucidating the regulatory mechanism of mRNA subcellular localization plays an important role in understanding the pathogenesis of human diseases, helping to further explain the etiology of related diseases at the molecular level and to develop corresponding drugs for related diseases. RNA fluorescence in situ hybridization (RNA-FISH) and high-throughput sequencing have been widely used to identify the distribution of mRNA in complicated cell spaces [7,8,9,10,11]. However, because traditional experiments are time-consuming and costly, better approaches to addressing this issue are required.

Artificial intelligence (AI) has advanced significantly over the last ten years, which has had a significant impact on the life sciences. AI is altering the way that life scientists conduct their research, particularly in the wake of AlphaFold 2’s successful prediction of the three-dimensional structure of proteins [12]. The most unexpected advancement in AI recently is ChatGPT (https://chat.openai.com/, accessed on 10 February 2023), which provided us the impression that the AI era of the life sciences is about to arrive. AI can assist us in a variety of analyses, such as assisting us with studying genome sequences [13], identifying new drugs [14], conducting comprehensive multiomics profile analyses [15], diagnosing diseases using medical images [16], and constructing multimodal medical knowledge maps [17], among others. AI has contributed significantly to numerous assessments of biological sequences [18,19,20,21,22,23]. Several online databases that may be used as training datasets for machine-learning models have been constructed using data from wet-lab experiments. Lin et al. constructed the RNA subcellular localization database, RNALocate v1.0 [24], in 2015, followed by an updated version named RNALocate v2.0 [25] in 2021, involving over 110,000 RNAs with 171 subcellular localizations in 104 species and providing a number of available data for in silico predictions of mRNA subcellular localization.

Many techniques for predicting mRNA subcellular localization have been proposed in recent years; however, their prediction performance still needs to be improved. RNATracker [26] was the first computational predictor for mRNA subcellular localization, which stacked two convolutional layers combined with a bi-directional long short-term memory (BiLSTM) layer and a self-attention module. Subsequently, some other solutions were proposed. Zhang et al. proposed iLoc-mRNA [27], in which support vector machine (SVM) was used as the classifier. However, both RNATracker and iLoc-mRNA are limited to predicting mRNA subcellular locations in *Homo sapiens*. Garg et al. developed mRNALoc [28] to predict the mRNA subcellular localization in eukaryotes, in which the feature information in the mRNA sequence is extracted using the pseudo K-tuple nucleotide composition (PseKNC) [29], and then a support vector machine-based model is constructed to perform the prediction. Based on LightGBM [30], Li et al. proposed SubLocEP [31], which is the best prediction model known to date.

However, we found that the algorithms mentioned above need to be improved in the following three aspects: (1) the prediction accuracy needs to be further improved, (2) some models have obvious over-fitting problems, and (3) the generalization ability of some algorithms also needs to be improved.

In this paper, we proposed DeepmRNALoc, a deep learning-based prediction model, and compared it to other existing models, demonstrating significant improvements in predicting eukaryotic mRNA subcellular localization.

## 2. Results

### 2.1. Model Training Based on Five-Fold Cross-Validation

Using the training set, the five-fold cross-validation results revealed that the *precision*, *recall*, *accuracy*, and *F-score* were as follows (Table 1): cytoplasm (0.777, 0.873, 0.873, and 0.822), endoplasmic reticulum (0.813, 0.516, 0.516, and 0.631), extracellular region (0.573, 0.326, 0.326, and 0.415), mitochondria (0.867, 0.897, 0.897, and 0.882), and nucleus (0.843, 0.856, 0.856, and 0.850).

By examining the prediction outcomes, we discovered that while the sample size of mitochondria was the smallest among the five classes, its performance was the highest among the five assessment indicators, suggesting that mitochondria were the most easily located subcellular type. The extracellular region had a small sample size, and its classification performance was the worst. The sample sizes for the cytoplasm and nucleus were the largest, and their classification performance was also the best. More samples were required to achieve a higher classification accuracy when using deep learning to predict the subcellular classification of eukaryotic mRNA.

### 2.2. Trained Model Evaluation Based on an Independent Dataset

The trained mode of DeepmRNALoc was validated by using the independent validation dataset, with the results showing that the precision, recall, accuracy, and F-score values were as follows (Table 2): cytoplasm (0.802, 0.895, 0.895, and 0.846), endoplasmic reticulum (0.816, 0.594, 0.594, and 0.688), extracellular region (0.603, 0.308, 0.308, and 0.407), mitochondria (0.931, 0.944, 0.944, and 0.937), and nucleus (0.857, 0.865, 0.865, and 0.861).

We demonstrated that DeepRNALoc does not exhibit over-fitting by comparing the results in Table 1 and Table 2, indicating that our model has a strong generalization ability.

### 2.3. Performance Comparisons of the DeepmRNALoc with Other Existing State-of-the-Art Predictors

**Figure 1:**
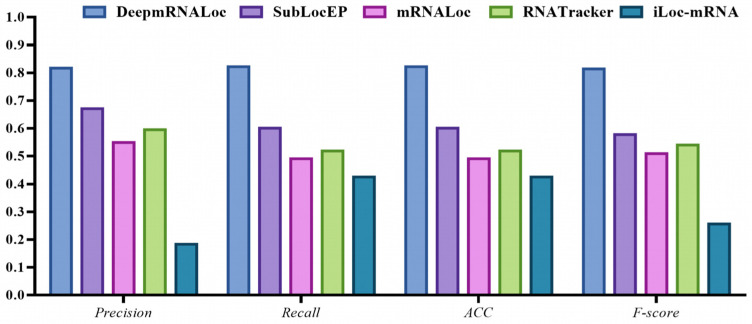
Performance comparison of DeepmRNALoc, SubLocEP, mRNALoc, iLoc-mRNA, and RNATracker under different evaluation indicators.

To further assess the performance of DeepmRNALoc, we compared DeepmRNALoc with other published state-of-the-art mRNA subcellular localization prediction models, including SubLocEP [31], mRNALoc [28], iLoc-mRNA [27], and RNATracker [26]. The results showed that DeepmRNALoc outperformed all the other models for all four evaluation metrics (Table 3 and Figure 1).

The experimental results revealed that our model performed significantly better than the other four models (Table 3). Our model scored more than 80% for all five evaluation indicators, while the other four models scored less than 70%. SubLocEP performed the best of the four models, while iLoc-mRNA performed the worst.

### 2.4. Trained Model Evaluation Based on Independent Human mRNA Data (Dataset 2)

In medicine, determining the subcellular localization of human mRNA is an important task, so it was necessary to select human mRNA data to evaluate the performance of our model. As shown in Table 3, DeepmRNALoc showed a better prediction performance than the other algorithms, followed by SubLocEP. Therefore, SubLocEP was selected to compare with our model to evaluate the performance of DeepRNALoc. The results showed that DeepRNALoc outperformed SubLocEP for all three types of human mRNA data, which proved that our model has a strong robustness and generalization ability (Table 4).

### 2.5. Performance Comparison of Various k-Value Combinations

**Figure 2:**
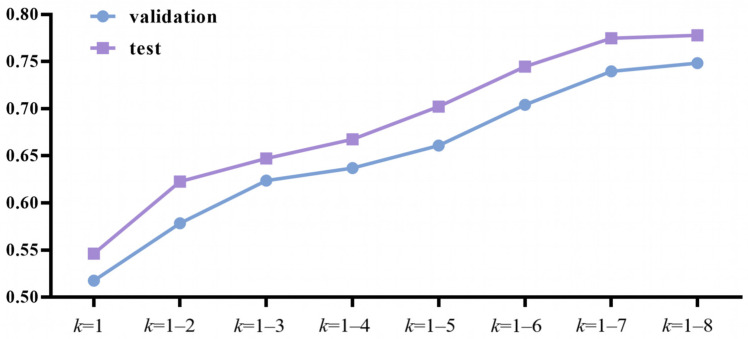
Accuracy with various *k* values.

When utilizing k-mer to extract feature information from mRNA sequences, the value of *k* influenced the prediction accuracy significantly. Taking into account the computational complexity and computing power available, we performed many calculation experiments with various *k* value combinations to find the best *k* value. We tried *k* = 1; *k* = 1–2; *k* = 1–3; *k* = 1–4; *k* = 1–5; *k* = 1–6; *k* = 1–7; and *k* = 1–8. The results showed that as the *k* value continued to increase, the prediction accuracy of the model also continued to increase (Figure 2). However, as *k* increased, so did the dimensionality of the input data. Due to computing power constraints, we limited the maximum value of k to 8, and obtained k-mer data with k ranging from 1 to 8.

### 2.6. Model Deployment, Web Server Construction, and Usage

**Figure 3:**
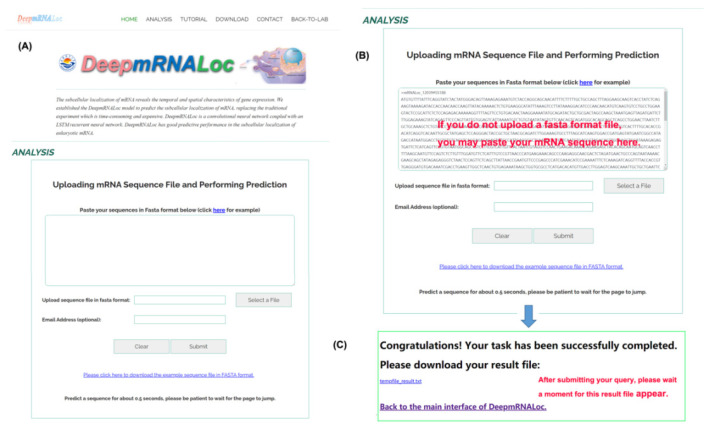
Screenshots of the DeepmRNALoc web server. (**A**) The web interface; (**B**) input data upload and information-filling interface; and (**C**) result download page.

To facilitate the use of our model for mRNA subcellular localization predictions by biomedical researchers, a trained model was deployed on a web server, which can be publicly accessed at http://www.peng-lab.org:8080/mRNA/ (accessed on 10 February 2023) (Figure 3A). Users can upload their sequence file in fasta format to the server or paste the sequence directly into the text box on the server interface (Figure 3B). The mRNA sequence submitted to the web server must be in fasta format, with the base characters of G, C, A, and T. Each query sequence must be at least 100 bp long. If the submitted sequence does not conform to the fasta format, the web server will notify the user with the message “your file did not pass the format check” and request the user to resubmit the sequence in the proper format. After clicking the submit button, the page will navigate to a new page where the user can download the result file (Figure 3C). If the user enters his email address (optional) in the text input box before submitting the query, the result file will be automatically emailed to the user.

## 3. Materials and Methods

### 3.1. Benchmark Datasets

#### 3.1.1. Dataset 1 (Training Set and Independent Validation Set)

To compare the performance of the models, we chose data used in previously published research [28,31] that was originally extracted from the RNALocate database [25]. Here, we named this dataset Dataset 1. According to their subcellular location in the cell, the samples of Dataset 1 were divided into five classes: cytoplasm (6376), endoplasmic reticulum (1426), extracellular region (855), mitochondria (421), and nucleus (5831).

It should be noted that the original Dataset 1 we downloaded contained two sets of data; one was a training set and the other was an independent validation set. However, we found that there was a statistically significant difference in the length of the mRNA sequences between these two groups of data (*t*-test, *p* < 0.001). We believe that it would have been unreasonable to use this independent validation set for model validation. To this end, we merged the two groups of datasets and then shuffled the merged dataset. Finally, a group of data was randomly selected from the shuffled dataset as the independent validation dataset (independent validation set), in which the sample size was equal to the original one, and the rest was used as the model-training dataset (training set).

#### 3.1.2. Dataset 2 (Independent Validation Dataset for Human mRNA Subcellular Localization)

Dataset 2 was derived from human mRNA subcellular localization data used in previous studies [27]. Because an incorrect subcellular localization may cause many human diseases, such as cancer and Parkinson’s syndrome, it is particularly important to predict the subcellular localization of human mRNA. For this reason, we specially selected Dataset 2 as an extra validation dataset to validate our model’s ability to predict the subcellular localization of human mRNA. There were three classes in the samples of Dataset 2, including the cytoplasm, endoplasmic reticulum, and nucleus.

Dataset 1 and Dataset 2 are available to the public in the Supplementary Materials.

### 3.2. Numerical Coding of mRNA Sequence Data

Because current machine-learning algorithms can only handle numerical data, the first step was to numerically encode the RNA sequence data. In many algorithms, a single numerical encoding method is employed to encode the mRNA sequences, e.g., one-hot [32], k-mer [33], and CGR (chaos-game representation [34]). In this study, we combined the k-mer and CGR methodologies to encode the RNA sequence data.

k-mer: In bioinformatics, k-mer is a common and useful numerical coding method for sequence data that represents the intrinsic information contained in a nucleotide sequence [35,36]. k-mer starts from the first base at the beginning of the sequence (S), moves *k* bases as the basic unit of the sliding window to the terminal base, and intercepts (*L−k*+1) nucleotide sequences of length *k*, where *L* is the length of the sequence. By calculating all the occurrence frequencies (Fs) of the k-tuple nucleotides in the whole sample sequence, a numeric vector is constructed [37,38]. When *k* is determined, the dimension of the corresponding numeric vector (sequence features) is 4*^k^*. The bigger the value of *k* is, the more information the numeric vector contains. However, as the value of *k* increases, the computational complexity will increase dramatically. Taking into account the computing power of our computer hardware, the *k* value was set to 1–8.

The occurrence frequency *F* was calculated as follows:

Nucleotide sequence $S=R_{1}R_{2}R_{3}\ldots R_{i}\ldots R_{L}\;R_{i}\in \left\{A,G,C,T\right\}$.

(1) $$F=\frac{n_{i}}{\Sigma_{i=1}^{4^{k}}n_{i}}=\frac{n_{i}}{L-k+1}$$

where *n_i_* denotes the number of occurrences of the *i*-th k-tuple nucleotide component in the mRNA sequence *S*, and *L* denotes the length of the sequence.

Chaos-Game Representation (CGR): The CGR approach can encode the information contained in a sequence into digital image information, and the deep-learning algorithm is particularly superior in image information processing, which prompted us to think of using CGR as the second method of encoding the sequence into numeric data. The sequence information was converted into a digital image as follows:

1. A square was generated;
2. The four different nucleotides were marked on each corner of the square;
3. The first point at the center point of the square was generated as the starting point;
4. A straight line was drawn from the center point of the square to the corner corresponding to the first nucleotide of the sample sequence, taking the midpoint of the line as the second point, and then another straight line was drawn from the second point to the corner corresponding to the second nucleotide of the sample sequence, taking the midpoint of the straight line as the third point, and so on, until the nucleotides of the sample sequence were used up [34,39,40,41].

The image obtained through the above steps was a binary image, with the position of the drawn point set to “1” and the position of no point set to “0”. By flattening this binary image, a numeric vector was obtained.

Finally, the concatenated numeric vector obtained from CGR and k-mer was fed into a neural network.

### 3.3. Network Architecture of DeepmRNALoc

The k-mer approach [42] was used to generate a numeric vector with a dimension of 87,380 (4^1^ + 4^2^ + 4^3^ + 4^4^ + 4^5^ + 4^6^ + 4^7^ + 4^8^), and the CGR methodology was used to obtain a digital image matrix (184 × 247) [34,39,40,41]. After flattening the matrix, a numeric vector with 45,448 dimensions was created for the digital image. Finally, the above two numeric vectors were concatenated, yielding a numeric vector with 132,828 dimensions as the input of DeepmRNALoc.

**Figure 4:**
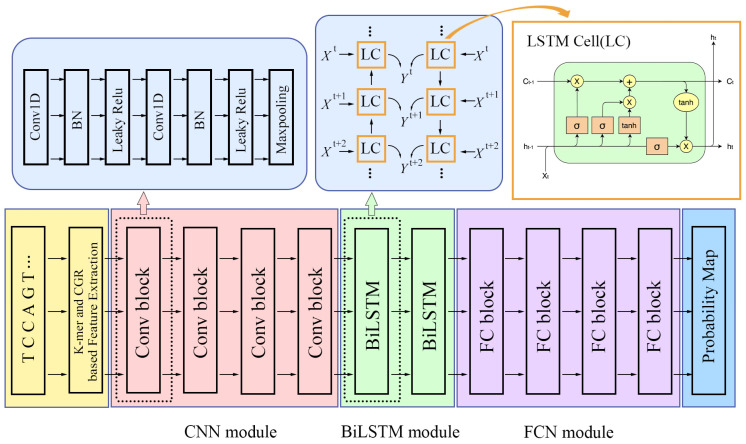
An illustration of DeepmRNALoc architecture. The CNN module contains four CNN blocks, with each CNN block consisting of two CNN layers. The BiLSTM module consists of two BiLSTM layers. The FCN module consists of four fully connected neural network layers.

DeepRNALoc is composed of three modules: a convolutional neural network (CNN) module, a BiLSTM module, and a fully connected neural network (FCNN) module. Each module contains multiple blocks. The architecture of DeepmRNALoc is shown in Figure 4.

In each CNN block, there are two CNN layers. The first CNN layer has a BN (batch normalization) layer and an activation function layer as its subsidiary layer. The second CNN layer has one more Maxpooling layer than the first CNN layer. The convolution kernel numbers in both convolutional layers for the first CNN block were 32; for the second, third, and fourth CNN blocks, the convolution kernel numbers in both convolutional layers were 64, 128, and 256, respectively. All kernel sizes in all the above CNN layers were set to 3 × 1, the convolution kernel strides were set to two, and the activation functions were set to LeakyReLU.

The BiLSTM module was made up of two BiLSTM layers, each with a LeakyReLU activation function.

The FCN module consisted of four fully connected layers, including an input layer, two hidden layers, and an output layer. The output dimensions of the input layer and the two hidden layers were all 512, and the output dimension of the output layer was five, which indicated that there were five classes of subcellular localization. The input layer and each hidden layer were followed by a batch normalization layer and a LeakyReLU activation function layer. On the output layer, we set SoftMax as the activation function, which assigned a probability value to each output class.

### 3.4. Performance Evaluation Criteria

When comparing the predictive performance of different models, most evaluation indicators can only show the predictive performance of the model from a specific aspect, so we used several statistical indicators, including accuracy (*ACC*, Equation (4)), *recall* (Equation (3)), *precision* (Equation (2)), and *F-score* (Equation (5)) [43,44,45]. The *ACC* indicates the prediction accuracy; however, when the sample size belonging to different classes in the data is uneven, ACC’s assessment conclusions may be questionable. *Recall* is the proportion of correctly predicted positive sets out of all positive sets. *Precision* represents the proportion of correctly predicted positive sets out of all predicted positive sets. The *F-score* is defined as the harmonic mean of the model’s *precision* and *recall*.

(2) $$Precison=\frac{TP}{TP+FP}$$

(3) $$Recall=\frac{TP}{TP+FN}$$

(4) $$ACC=\frac{TP+TN}{TP+TN+FP+FN}$$

(5) $$F\text{-}score=\frac{2\times Precison*Recall}{Precison+Recall}$$

*TP* and *FN* indicate that when the true label of the sample is positive, the prediction labels are positive and negative, respectively. *TN* and *FP* indicate that when the real label of the sample is negative, the prediction labels are negative and positive, respectively.

### 3.5. Five-Fold Cross-Validation for Model Validation and Over-Fitting Issue

Cross-validation can improve a model’s generalization ability, which is a key criterion for determining if a model is excellent or bad. For this reason, DeepmRNALoc used cross-validation during the model-training process. Typically, models are built using a ten- or five-fold cross-validation strategy [46]. Cross validation slices and combines samples into different training sets and test sets. The training set is used to train the model, while the test set is used to evaluate the model. In the training process, the predicted average value of the metrics obtained from each test set is calculated, and the result then guides the next round of model training. In DeepmRNALoc, a five-fold cross-validation strategy was adopted.

A model’s evaluation is based on its evaluation indicators/metrics, such as accuracy and precision, as well as whether it exhibits over-fitting and whether it has excellent generalizability. Over-fitting is a modeling error that occurs when the function is too close to a limited set of data points. Therefore, the model is only applicable to its initial dataset, and not to any other dataset.

The method to test whether the model is over-fitted is to use independent datasets for model validation. If the classification performance for the independent datasets is significantly worse than the accuracy attained during the model-training procedure, over-fitting is likely to be present. If the two results of accuracy are comparable, there is no over-fitting in the model. If the independent verification dataset’s prediction performance is significantly better, it demonstrates that the trained model has high generalization capabilities and does not exhibit over-fitting.

## 4. Discussion

In general, the deep-learning algorithm performed better when the data size was big enough. RNATracer has performed pioneering work. On the one hand, RNATracer is the first work of mRNA subcellular localization prediction. On the other hand, it uses a deep-learning algorithm to address this issue. The RNATracer model consists of CNN, LSTM, and fully connected layers. Unfortunately, the mRNA sequence information coding used is the one-hot coding method, and this is the main reason for the poor classification performance of RNATracer.

ILoc-mRNA adopted the k-mer coding method, which is superior to one-hot coding, and it adopted the traditional machine-learning method.

As for mRNALoc and SubLocEP, the traditional machine-learning algorithm was adopted, and the input sequence data coding also used single-mode data, so they did not show a superior performance in model prediction.

DeepmRNAloc does not simply use a one-hot coding method to numerically encode mRNA sequences, nor does it extract features using single-modal information. We used a two-step feature extraction method. The k-mer and CGR approaches were used in the first feature extraction procedure. On the one hand, the mRNA sequences are encoded numerically in this step, and on the other hand, this step is also the process of feature extraction. By analyzing this feature extraction step, it can be seen that we in fact converted the sequence information into two-modal information using k-mer and CGR, fused (merged) the two-modal information, and then sent it to CNN modules to perform the second step of feature extraction. We believe this is one of the reasons why our model outperformed other existing models.

Using only fully connected networks may lead to two major problems: (1) the network has too many trainable parameters, resulting in a low training efficiency, and (2) a large number of trainable parameters can easily lead to model overfitting. Because CNN is very good at solving the difficulties mentioned above, it was used in DeepmRNALoc to extract feature information from the input data. A network structure similar to VGGNet [47] was used in the CNN module of DeepmRNALoc.

Several methods for mRNA subcellular localization have been proposed in recent years [26,27,28], e.g., both iLoc-mRNA and mRNALoc use SVM as the classifier, while DeepmRNALoc uses a deep neural network as the classifier. Many studies have shown that deep neural networks are more robust and often have a better predictive performance than SVMs [48,49]. On the other hand, other models have adopted single-mode information-coding methodologies, and we believe that the information obtained by single-mode coding is incomplete. To this end, we used dual-mode information coding, so compared with other methods, our coding process may lose less information. Some models even use the one-hot coding method, which is obviously the coding method with the least information. This is because the genetic information of biological sequences is included in the sequence arrangement, so a simple coding method based on Euclidean distance will lose a lot of the genetic coding information. Therefore, the dual-mode coding we adopted made our model’s over-fitting lower than that of other models, and our model’s generalization ability was significantly stronger than that of other models.

DeepmRNALoc and RNATracker are both deep neural network-based mRNA subcellular localization models; however, RNATracker can only predict human mRNA subcellular localization, whereas DeepmRNALoc can predict eukaryotic mRNA subcellular localization.

As the subcellular localization of human mRNA is the most important category, we used Dataset 2, in which all the mRNA sequences were from humans, to validate our model again. Table 4 shows that our model had an excellent performance, and its prediction accuracy was significantly higher than that of the current SOTA algorithm SubLocEP, indicating that DeepRNALoc has no over-fitting and has a better generalization ability.

## 5. Conclusions

In this study, we proposed DeepmRNALoc, a novel tool for predicting eukaryotic mRNA subcellular localization using a deep neural network, which offers significant advantages over previously reported techniques. In addition, the pre-trained model-based DeepmRNALoc was deployed on the web server and is publicly available to biomedical researchers.

Among the five subcellular classes, two of them (extracellular region and endoplasmic reticulum) had a small sample size, and the prediction performance of our deployed model may be lower for these two classes, which is a limitation of DeepmRNALoc. To compensate for this shortcoming, we will constantly upgrade the model deployed on the web server to increase its prediction accuracy as more data are collected.

## Figures and Tables

**Table 1:** The performance of DeepmRNALoc on the training set (in Dataset 1) using 5-fold cross-validation.

	Evaluation Metrics			
Location	*Precision*	*Recall*	*ACC*	*F-Score*
Cytoplasm	0.777	0.873	0.873	0.822
Endoplasmic reticulum	0.813	0.516	0.516	0.631
Extracellular region	0.573	0.326	0.326	0.415
Mitochondria	0.867	0.897	0.897	0.882
Nucleus	0.843	0.856	0.856	0.850

**Table 2:** The performance of DeepmRNALoc on the independent validation set (in Dataset 1).

	Evaluation Metrics			
Location	*Precision*	*Recall*	*ACC*	*F-Score*
Cytoplasm	0.802	0.895	0.895	0.846
Endoplasmic reticulum	0.816	0.594	0.594	0.688
Extracellular region	0.603	0.308	0.308	0.407
Mitochondria	0.931	0.944	0.944	0.937
Nucleus	0.857	0.865	0.865	0.861

**Table 3:** Performance comparison of the five models based on the training set (Dataset 1).

Model	*Precision*	*Recall*	*ACC*	*F-Score*
DeepmRNALoc	0.817	0.822	0.822	0.814
SubLocEP	0.671	0.601	0.601	0.578
RNATracker	0.595	0.519	0.519	0.540
mRNALoc	0.549	0.491	0.491	0.509
iLoc-mRNA	0.183	0.425	0.425	0.256

**Table 4:** Comparison of accuracy between DeepmRNALoc and SubLocEP based on Dataset 2.

	DeepmRNALoc	SubLocEP
Cytoplasm	0.923	0.883
Endoplasmic reticulum	0.811	0.630
Nucleus	0.807	0.426

## Data Availability

All the data sets and source code are publicly available through the GitHub (https://github.com/Thales-research-institute/DeepmRNALoc) (accessed on 10 February 2023).

## Supplementary Materials

The following supporting information can be downloaded at: https://github.com/Thales-research-institute/DeepmRNALoc.
